# Dietary linoleic acid supplementation protects against obesity-induced microglial reactivity in mice

**DOI:** 10.1038/s41598-024-56959-6

**Published:** 2024-03-19

**Authors:** Lucas Jantzen, Stéphanie Dumontoy, Bahrie Ramadan, Christophe Houdayer, Emmanuel Haffen, Aziz Hichami, Naim Akhtar Khan, Vincent Van Waes, Lidia Cabeza

**Affiliations:** 1 Université de Franche-Comté, UMR INSERM 1322 LINC, 19, Rue Ambroise Paré, 25000 Besançon Cedex, France; 2 Université de Franche-Comté, UMR INSERM 1322 LINC, service de psychiatrie de l’adulte, CIC-1431 INSERM, CHU de Besançon, 25030 Besançon, France; 3 Physiologie de la Nutrition & Toxicologie (NUTox), UMR UB/Institut Agro/INSERM U1231, Lipides, Nutrition & Cancer, LABEX-LipStick, Université de Bourgogne, Dijon, France

**Keywords:** Neuroscience, Obesity

## Abstract

We investigated whether linoleic acid (LA) supplementation could modulate emotional behavior and microglia-related neuroinflammation. For that, male mice of C57BL/6J genetic background fed either a high-fat diet (HFD) or a standard diet (STD) for 12 weeks, were treated with a vehicle or LA solution for 5 weeks before being evaluated for emotional behavior using a battery of behavioral tests. The animals were subsequently sacrificed and their brains collected and processed for immunofluorescence staining, targeting microglia-specific calcium-binding proteins (IBA-1). Neuroinflammation severity was assessed in multiple hypothalamic, cortical and subcortical brain regions. We show an anxio-depressive-like effect of sustained HFD feeding that was neither alleviated nor worsened with LA supplementation. However, increased IBA-1 expression and microgliosis in the HFD group were largely attenuated by LA supplementation. These observations demonstrate that the anti-neuroinflammatory properties of LA are not restricted to hypothalamic areas but are also evident at the cortical and subcortical levels. This study discloses that neuroinflammation plays a role in the genesis of neuropsychiatric disorders in the context of obesity, and that LA supplementation is a useful dietary strategy to alleviate the impact of obesity-related neuroinflammation.

## Introduction

The multifactorial etiology of obesity obscures the underlying biological mechanisms^1^. Besides genetic vulnerability^2,3^, a large number of obesity cases in Western countries are the consequence of malnutrition, i.e., overconsumption of fat-rich food. Prolonged high-fat diets lead to the emergence of metabolic, cardiac, respiratory and endocrinal comorbidities^4,5^, and obesity has been correlated with neuropsychiatric disorders such as anxiety and depression^6,7^ in clinical^8,9^ and preclinical studies^10–13^.

Obesity has drawn much scientific attention since the crucial role of gut microbiota in human health was demonstrated^14^. Increased concentrations of short-chain fatty acids (SCAFs) released by the gut microbiota have been reported in individuals suffering from obesity^15^. Under physiological conditions, these products of colon microbial food fermentation induce the release of anorexigenic hormones into the peripheral circulation^16^, thus increasing circulating concentrations of leptin and insulin^17^. Since SCAFs can cross the intestinal epithelium to reach the portal vein and stimulate the vagal nerve, they may influence the homeostatic balance regulating individuals’ appetite. Additionally, SCAFs modulate the immune response. They can reduce peripheral inflammation by inhibiting the production of pro-inflammatory cytokines^18^, and participate in the maturation of the microglia in the central nervous system (CNS)^19^. Subsequently, the abnormally elevated circulating SCAF concentrations in obesity correlate with higher levels of lipopolysaccharides (LPS). Elevated circulating SCAF concentrations and increased levels of LPS therefore correlate with the severity of the resulting systemic inflammation^20^. Additionally, the expression of gustatory receptors, which are sensitive to circulating LPS levels, is downregulated in individuals suffering from obesity, consequently leading to altered gustatory perception that might motivate food overconsumption (for a review, see^21^).

Since it was first observed in the hypothalamus^22^, extensive research has focused on obesity-induced neuroinflammation. Thus, different mechanisms have been described in an attempt to unravel its role in the pathogenesis of obesity^23,24^; however, further investigations are needed to understand how this phenomenon affects the hypothalamus and other surrounding regions.

Linoleic acid (LA, C18:2n-6) is an essential omega-6 (ω-6) polyunsaturated fatty acid present in vegetable oils, nuts, seeds, meat, and eggs^25^. Like other ω-6 fatty acids, LA participates in maintaining the cellular membrane structure and preserving nerve blood flow through the formation of prostaglandins. As a precursor of eicosanoids, it modulates vital functions (e.g. pulmonary function), vascular tone, and the inflammatory response (for a review, see^26^). Linoleic acid has become ubiquitous in Western diets due to the incorporation of high-LA soybean and corn oils, representing approximately 7% of the intake of daily calories (for a review, see^25^). Thus, high-fat regimens’ pathogenicity seems to depend more on the ratio between ω-6 and ω-3 fatty acids (e.g., alpha-linolenic acid)^27^, and the present fraction of saturated fatty acids.

Preclinical studies have demonstrated that the hypothalamic microglial-related inflammatory response induced by short-term high-fat regimens can be rescued by LA^28^. However, neuroinflammation chronicity might limit the anti-inflammatory efficacy of LA. We, therefore, propose to evaluate the overall brain effects of LA supplementation on obesity-related neuroinflammation induced by feeding a diet high in saturated fat. We hypothesize that LA exerts anti-neuroinflammatory effects beyond the hypothalamic region and contributes to alleviating emotional disturbances.

## Results

**Figure 1:**
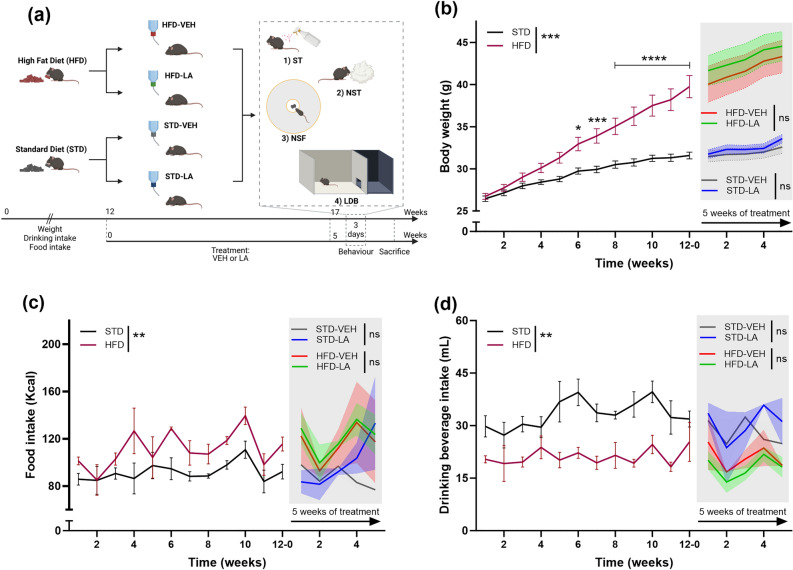
Experimental design and animal monitoring. (**a**) Animals were fed either a high-fat (HFD) or a standard (STD) diet for 12 weeks before being treated with vehicle (VEH) or a 0.2% linoleic acid (LA) solution for 5 weeks. The animal’s body weight and consummatory behavior were monitored during the entire experiment. The effect of the regimen and LA supplementation on emotional behavior was evaluated during the last week of the experiment through a battery of tests: the splash test (ST), the nestlet shredding test (NST), the novelty-supressed feeding (NSF) task, and the light–dark box (LDB) test. Animals were then sacrificed for IBA-1 immunofluorescence staining. Created with *BioRender.com*. (**b**) All animals progressively gained weight during the experiment, and mice fed a HFD weighed significantly more than their control counterparts (STD) from the sixth week of the differential regimen. However, 5 weeks of LA supplementation did not affect the body weight of animals in either the HFD or the STD group. (**c**) A general effect of the diet on the weekly food intake (Kcal) was evidenced, with HFD mice consuming significantly more calories than control animals. Besides, LA supplementation did not significantly impact food intake in either obese (HFD-LA vs. HFD-VEH) or non-obese animals (STD-LA vs. STD-VEH). (**d**) Sustained fat intake significantly influenced the weekly beverage intake in mice, with HFD individuals drinking a significantly lower volume than control animals. However, LA supplementation did not influence this behavior in either obese (HFD-LA vs. HFD-VEH) or non-obese animals (STD-LA vs. STD-VEH). Values plotted are mean ± SEM (n = 10 per group). *, p < 0.05; **, p < 0.01; ***, p < 0.001; ****, p < 0.0001; ns, not significant.

The timeline of the experimental design is presented in Fig. 1a. The following four main experimental groups were used (see “Methods” section for details): (1) a control group of mice fed a standard diet and treated with vehicle solution (STD-VEH); (2) a group of mice fed a STD and treated with LA solution (STD-LA); (3) a group of mice fed a high-fat diet and treated with VEH solution (HFD-VEH); and (4) a group of mice fed a HFD and treated with LA solution (HFD-LA).

### Body weight and consummatory behavior

Mice maintained on an HFD significantly increased their body mass over 12 weeks (mean weight (g) ± SEM: 39.75 ± 1.32) compared to those fed a STD (31.59 ± 0.40) [RMA: main effect of diet, F_1,38_ = 18.0, p = 0.0001; mean effect of time, F_11,418_ = 178.9, p < 0.0001; diet × time interaction, F_11,418_ = 34.0, p < 0.0001] (Fig. 1b). Animals from the HFD group consumed significantly more calories than control animals (food intake per week (kcal) ± SEM, HFD: 111.30 ± 4.36; STD: 91.72 ± 2.20) [diet: F_1,6_ = 24.2, p = 0.003; time: F_11,66_ = 2.7, p = 0.007; diet × time interaction, F_11,66_ = 0.7, p = 0.70], and their drinking intake was lower (beverage intake per week (mL) ± SEM, HFD: 21.15 ± 2.35; STD: 33.33 ± 3.96) [diet: F_1,6_ = 23.6, p = 0.003; time: F_11,66_ = 1.6, p = 0.11; diet × time interaction, F_11,66_ = 1.1, p = 0.41]. This consummatory behavior remained unaltered during the LA supplementation period (food intake, HFD-VEH: 115.70 ± 6.74; HFD-LA: 120.80 ± 6.29; STD-VEH: 87.72 ± 4.12; STD-LA: 98.79 ± 9.45; treatment intake, HFD-VEH: 20.97 ± 1.57; HFD-LA: 18.10 ± 1.38; STD-VEH: 27.93 ± 1.67; STD-LA: 30.57 ± 2.09) [food: F_3,4_ = 1.8, p = 0.29; time: F_4,16_ = 1.6, p = 0.21; food x time interaction, F_12,16_ = 0.8, p = 0.66; treatment: F_3,4_ = 2.6, p = 0.19; time: F_4,16_ = 3.1, p = 0.043; treatment x time interaction, F_12,16_ = 0.5, p = 0.88] (Fig. 1c, d).

### Behavioral evaluation

**Figure 2:**
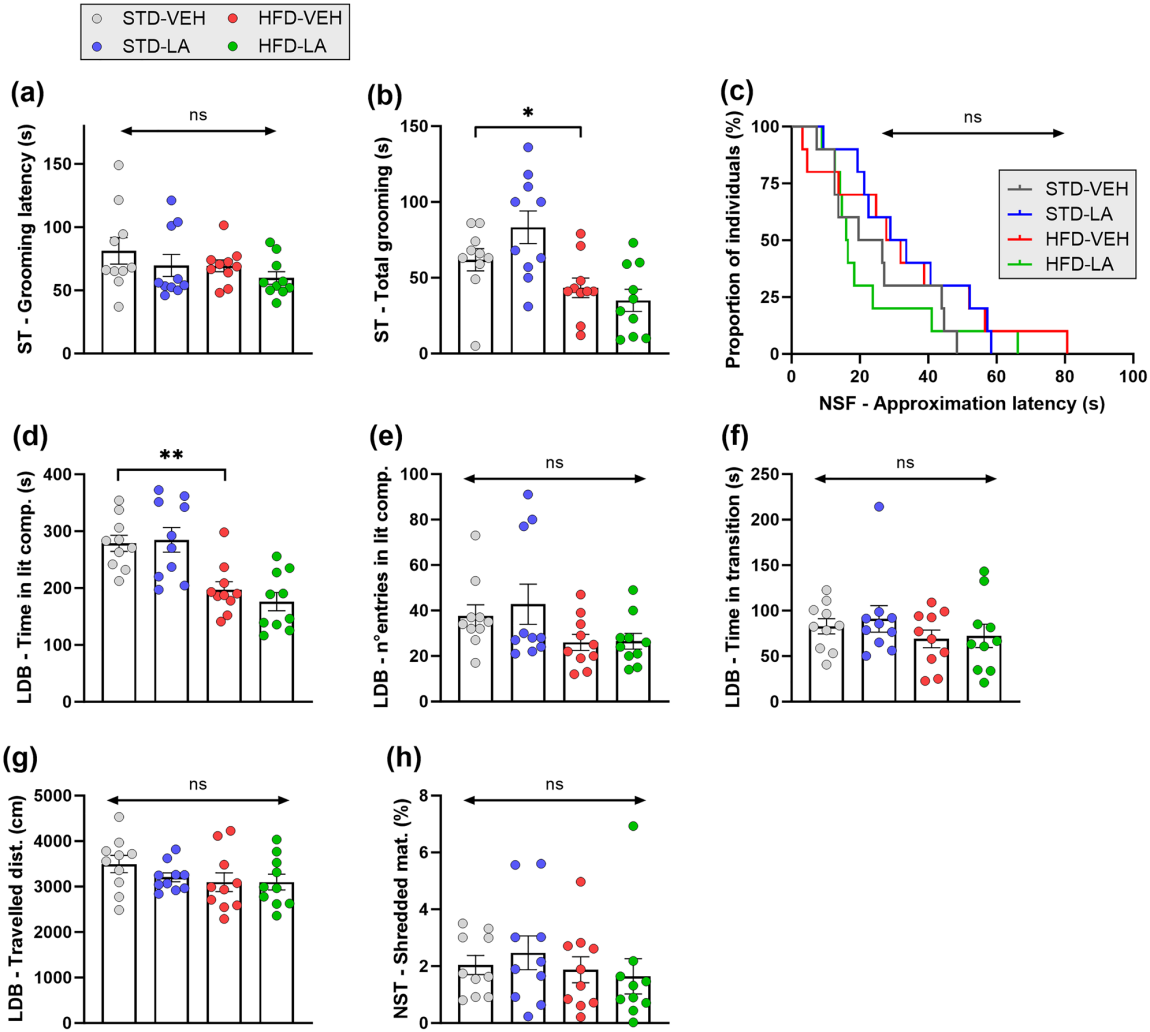
Effect of 5 weeks of linoleic acid (LA) supplementation on sustained high-fat diet (HFD)-induced emotional alterations. (**a**) Even if the latency of the first grooming event in the splash test (ST) was similar for both experimental groups, (**b**) a depressive-like phenotype consequent to a sustained HFD was evidenced by ST, with obese animals (HFD-VEH) spending less time grooming themselves than those fed a standard diet (STD-VEH). (**a**, **b**) Linoleic acid supplementation affected the behavior of neither obese mice (HFD-LA) nor non-obese animals (STD-LA) in the ST. (**c**) The behavioral assessment using the novelty-suppressed feeding (NSF) task revealed no significant differences in emotionality among the experimental groups; all animals exhibited similar latencies in approaching the available food. (**d**–**g**) The light–dark box (LDB) test revealed the emergence of an anxious-like phenotype consequent to a sustained HFD, with obese animals (HFD-VEH) spending less time in the lit compartment than their control counterparts (STD-VEH) (**d**). This emotional alteration was not reflected in the other measured parameters, such as the frequency of visiting the lit compartment (**e**), the time in the transition area (**f**), and the traveled distance (**g**). No behavioral effect of 5 weeks of LA supplementation was observed in the LDB test (**d**–**g**), irrespective of the dietary regimen. (**h**) Sustained HFD did not induce the emergence of repetitive, compulsive-like behavior in mice, as evidenced in the nestlet shredding test (NST), and no impact of LA supplementation was revealed, irrespective of the regimen. Values are mean ± SEM (n = 10 per group). *, p < 0.05; **, p < 0.01; ns, not significant.

We first evaluated the impact of feeding mice a HFD for 17 weeks on their emotional behavior (STD-VEH vs. HFD-VEH). We confirmed the emergence of an anxio-depressive-like phenotype consequence of a prolonged HFD^10,11,29^. Animals in both conditions started grooming at similar latencies (mean grooming latency (s) ± SEM; STD-VEH: 81.22 ± 10.49; HFD-VEH: 72.23 ± 5.63) [H_3,40_ = 4.3, p = 0.23; Z = 0.076, p = 0.94] (Fig. 2a), but 17 weeks of the HFD significantly reduced the time mice spent grooming themselves in the splash test (ST) (total grooming time (s) ± SEM; STD-VEH: 61.90 ± 7.29; HFD-VEH: 43.40 ± 6.42) [KW, H_3,40_ = 13.2, p = 0.004; *post-hoc* MWU, Z = 2.05, p = 0.041] (Fig. 2b). Contrary to expectations, HFD-VEH and STD-VEH mice approached the food in the novelty-supressed feeding (NSF) task at comparable latencies (latency (s) ± SEM; STD-VEH: 25.10 ± 4.79; HFD-VEH: 32.80 ± 7.74) [GBW: Χ^2^ = 0.57, p = 0.451] (Fig. 2c). Furthermore, HFD-VEH animals showed typical anxious behavior in the light-dark box (LDB) test, spending significantly less time in the lit compartment (time in lit compartment (s) ± SEM; HFD-VEH: 197.15 ± 14.03) than their SDT-VEH counterparts (278.67 ± 14.20) [H_3,40_ = 20.0, p = 0.000; Z = 3.10, p = 0.0019] (Fig. 2d). However, no effect of the regimen was evidenced on the frequency of visiting the lit compartment (entries into lit compartment ± SEM; HFD-VEH: 26.00 ± 3.57; STD-VEH: 37.70 ± 4.84) [H_3,40_ = 6.0, p = 0.11] (Fig. 2e), the time spent in the transition zone (time in transition zone (s) ± SEM; HFD-VEH: 69.67 ± 9.72; STD-VEH: 82.81 ± 8.26) [H_3,40_ = 1.7, p = 0.63] (Fig. 2f), and the total distance traveled during the test (traveled distance (cm) ± SEM: STD-VEH: 3494.12 ± 188.37; HFD-VEH: 3096.50 ± 207.08) [H_3,40_ = 3.6, p = 0.30] (Fig. 2g). Concerning the nestlet shredding test (NST) evaluation, no differences were found between the experimental conditions (shredded material (%) ± SEM; STD-VEH: 2.04 ± 0.34; HFD-VEH: 1.87 ± 0.46) [H_3,40_ = 3.3, p = 0.34] (Fig. 2h).

We secondly studied the effect of 5 weeks of LA supplementation, starting from the 12th week of the differential regimen, on the animals’ behavior. Contrary to expectations, no differences between the experimental groups were found, neither for HFD mice (HFD-LA: ST, grooming time: 35.10 ± 7.28; grooming latency: 59.78 ± 4.88; NSF, approach latency: 22.80 ± 5.58; LDB, time in lit comp.: 176.06 ± 15.94; entries into lit comp.: 26.50 ± 3.43; time in transition: 72.28 ± 12.77; traveled distance: 3098.45 ± 172.83; NST, shredded material: 1.64 ± 0.62) [HFD-VEH vs. HFD-LA: Χ^2^ = 0.831, and all p-values > 0.1] nor for their STD counterparts (STD-LA: ST, grooming time: 83.30 ± 10.77; grooming latency: 69.67 ± 8.73; NSF, approach latency: 33.90 ± 5.44; LDB, time in lit comp.: 284.98 ± 21.67; entries into lit comp.: 42.80 ± 8.81; time in transition: 90.88 ± 14.59; traveled distance: 3204.34 ± 98.62; NST, shredded material: 2.47 ± 0.60) [STD-VEH vs. STD-LA: Χ^2^ = 1.46, and all p-values > 0.1] (Fig. 2a–h).

### Microglia reactivity evaluation through IBA-1 expression

Microglial reactivity was assessed through ionized calcium-binding adapter molecule 1 (IBA-1) staining. IBA-1 specifically targets microglia in the brain and is known to be upregulated during the activation of these cells. Microglial reactivity was evaluated in a selection of 6 hypothalamic regions (in rostral-to-caudal order: paraventricular nucleus—PVN; peduncular part of the lateral hypothalamus—PLH; ventromedial hypothalamus—VMH; lateral hypothalamus—LH; subthalamic nucleus—STN; parasubthalamic nucleus—PSTN), 5 cortical areas (prelimbic—PL; infralimbic—IL; orbitofrontal—OFC; anterior cingulate—ACC; anterior insular—aIC) and 9 subcortical regions (dorsolateral—DLS and dorsomedial—DMS striatum; dorsal region of the bed nucleus of the stria terminalis—dBNST; central—CeA and basolateral—BLA amygdala; dorsal hippocampus—dHipp; lateral habenula—LHb; ventral posteromedial thalamic nucleus—VPM; parvicellular part of the ventral posterior thalamic nucleus—VPPC). The Bregma coordinates, the microglial density values for each brain region evaluated and the statistical comparison between experimental conditions are detailed in Table 1. The IBA-1 relative fluorescence intensity values and the statistical comparisons between experimental conditions are presented in Table 2.

**Table 1:** Data of IBA-1 positive cells density (microglial density) and statistical comparisons between experimental conditions and brain regions of interest (ROI).

ROI	Microglial density (mean n° IBA-1^+^ cells/mm^2^ ± SEM)						KW—*H*; *post-hoc* MWU—*Z*		
	*Bregma* coord. (mm)	STD-VEH	STD-LA	HFD-VEH	HFD-LA	n	STD-VEH vs. STD-LA	STD-VEH vs. HFD-VEH	HFD-VEH vs. HFD-LA
Hypothalamic regions									
PVN	0.26–0.02 aB	213.66 ± 13.20	213.36 ± 10.55	290.38 ± 12.39	214.95 ± 7.58	29	H_3,29_ = 15.0, **p = 0.001**		
							Z = − 0.7, p = 0.49	Z = − 2.8, **p = 0.005**	Z = 3.1, **p = 0.002**
PLH	1.06–1.34 pB	189.07 ± 7.35	195.19 ± 6.21	242.31 ± 6.56	185.32 ± 8.37	28	H_3,28_ = 15.6, **p = 0.001**		
							Z = − 0.7, p = 0.48	Z = − 3.1, **p = 0.002**	Z = 3.1, **p = 0.002**
VMH	1.70–1.94 pB	229.57 ± 10.14	233.21 ± 7.96	306.35 ± 8.78	254.86 ± 6.28	29	H_3,29_ = 17.5, **p = 0.000**		
							Z = − 0.3, p = 0.77	Z = − 3.2, **p = 0.002**	Z = 2.8, **p = 0.005**
LH	2.06–2.30 pB	237.91 ± 5.80	250.47 ± 9.09	325.19 ± 14.85	248.69 ± 11.14	29	H_3,29_ = 15.8, **p = 0.001**		
							Z = − 0.9, p = 0.35	Z = − 3.3, **p = 0.001**	Z = 3.0 , **p = 0.003**
STN	2.06–2.30 pB	309.96 ± 8.83	298.33 ± 15.56	462.10 ± 28.25	309.89 ± 9.37	29	H_3,29_ = 17.0, **p = 0.000**		
							Z = 0.7, p = 0.49	Z = − 3.4, **p = 0.001**	Z = 3.1, **p = 0.002**
PSTN	2.06–2.30 pB	325.71 ± 11.12	337.97 ± 16.49	472.81 ± 13.73	357.33 ± 19.37	27	H_3,27_ = 16.0, **p = 0.001**		
							Z = − 0.5, **p = 0.61**	Z = − 3.1, **p = 0.002**	Z = 3.0 , **p = 0.003**
Cortical areas									
PL	1.94–1.70 aB	235.10 ± 7.68	248.41 ± 5.72	296.24 ± 3.72	235.57 ± 6.37	30	H_3,30_ = 18.6, **p = 0.000**		
							Z = − 1.6, p = 0.11	Z = − 3.4, **p = 0.001**	Z = 3.2, **p = 0.001**
IL	1.94–1.70 aB	225.29 ± 8.75	232.91 ± 3.83	291.88 ± 7.72	230.63 ± 8.34	30	H_3,30_ = 16.9, **p = 0.000**		
							Z = − 0.8, p = 0.42	Z = − 3.3, **p = 0.001**	Z = 3.2, **p = 0.001**
OFC	1.94–1.70 aB	266.86 ± 5.35	259.20 ± 6.35	317.25 ± 4.27	264.50 ± 4.59	30	H_3,30_ = 18.0, **p = 0.000**		
							Z = 1.0, p = 0.30	Z = − 3.4, **p = 0.001**	Z = 3.2, **p = 0.001**
ACC	1.10–0.98 aB	230.62 ± 14.58	250.00 ± 5.32	273.86 ± 6.76	217.20 ± 10.85	28	H_3,28_ = 11.0, **p = 0.011**		
							Z = − 0.9, p = 0.36	Z = − 2.2, **p = 0.031**	Z = 2.8, **p = 0.005**
aIC	0.26–0.02 aB	300.03 ± 9.49	318.94 ± 15.61	351.29 ± 9.81	331.52 ± 7.54	29	H_3,29_ = 9.1, **p = 0.028**		
							Z = − 0.9, p = 0.35	Z = − 2.9, **p = 0.003**	Z = 1.8, p = 0.07
Subcortical regions									
DMS	1.10–0.98 aB	246.79 ± 8.84	242.62 ± 14.26	288.46 ± 6.47	235.04 ± 9.46	28	H_3,28_ = 12.3, **p = 0.006**		
							Z = 0.3, p = 0.80	Z = − 2.8, **p = 0.005**	Z = 2.8, **p = 0.004**
DLS	1.10–0.98 aB	270.83 ± 12.57	271.98 ± 7.51	315.77 ± 12.57	293.75 ± 13.13	28	H_3,28_ = 8.0, **p = 0.045**		
							Z = − 0.2, p = 0.85	Z = − 2.3, **p = 0.024**	Z = 0.71, p = 0.48
dBNST	0.26–0.02 aB	209.70 ± 6.85	204.16 ± 9.42	286.20 ± 6.62	236.54 ± 6.51	29	H_3,29_ = 20.9, **p = 0.000**		
							Z = 0.3, p = 0.73	Z = − 3.4, **p = 0.001**	Z = 3.1, **p = 0.002**
dHipp	1.70–1.94 pB	330.90 ± 11.52	333.75 ± 13.23	419.78 ± 14.34	345.10 ± 5.57	29	H_3,29_ = 15.6, **p = 0.001**		
							Z = − 0.3, p = 0.73	Z = − 3.1, **p = 0.002**	Z = 2.1, **p = 0.04**
LHb	1.70–1.94 pB	291.87 ± 12.86	293.03 ± 7.09	348.31 ± 25.37	286.56 ± 12.69	29	H_3,29_ = 7.0, p = 0.073		
BLA	1.06–1.34 pB	322.64 ± 14.54	315.45 ± 10.04	416.04 ± 17.94	353.95 ± 15.25	29	H_3,29_ = 15.6, **p = 0.001**		
							Z = 0.8, p = 0.42	Z = − 3.2, **p = 0.002**	Z = 1.8, p = 0.07
CeA	1.06–1.34 pB	287.64 ± 17.71	258.29 ± 8.92	373.61 ± 17.62	257.40 ± 17.09	29	H_3,29_ = 15.7, **p = 0.001**		
							Z = 1.5, p = 0.13	Z = − 2.7, **p = 0.006**	Z = 2.8, **p = 0.004**
VPM	2.06–2.30 pB	252.81 ± 7.58	248.19 ± 8.85	342.91 ± 16.00	259.90 ± 4.00	29	H_3,29_ = 15.8, **p = 0.001**		
							Z = 0.6, p = 0.56	Z = − 3.2, **p = 0.002**	Z = 3.0, **p = 0.003**
VPPC	2.06–2.30 pB	239.20 ± 10.60	255.05 ± 9.55	368.39 ± 18.39	264.02 ± 12.21	29	H_3,29_ = 18.2, **p = 0.000**		
							Z = − 1.0, p = 0.32	Z = − 3.4, **p = 0.001**	Z = 3.1, **p = 0.002**

Significant values are in bold.

Bregma coordinates (mm) for sections selection: anterior to Bregma—aB; posterior to Bregma—pB. Food regimen: standard diet—ST; high-fat diet—HFD. Treatment: vehicle—VEH; linoleic acid supplementation—LA. Data are means ± SEM. Paraventricular nucleus of the hypothalamus—PVN, peduncular part of the lateral hypothalamus –PLH, ventromedial hypothalamus—VMH, lateral hypothalamus—LH, subthalamic nucleus of the hypothalamus—STN, parasubthalamic nucleus of the hypothalamus—PSTN, prelimbic cortex—PL, infralimbic cortex—IL, orbitofrontal cortex—OFC, anterior cingulate cortex—ACC, anterior insular cortex—aIC, dorsomedial striatum—DMS, dorsolateral striatum—DLS, dorsal region of the bed nucleus of the stria terminalis—dBNST, dorsal hippocampus—dHipp, lateral habenula—LHb, basolateral amygdala—BLA, central amygdala—CeA, ventral posteromedial thalamic nucleus—VPM, and parvicellular part of the ventral posterior thalamic nucleus—VPPC. n: sample size.

**Table 2:** Data of IBA-1 fluorescence intensity relative to the control group (standard diet-vehicle—STD-VEH; 100%) and statistical comparisons between experimental conditions and brain regions of interest (ROI).

ROI	IBA-1 relative fluorescence intensity (mean % ± SEM)				KW—*H*; *post-hoc* MWU—*Z*		
	STD-LA	HFD-VEH	HFD-LA	n	STD-VEH vs. STD-LA	STD-VEH vs. HFD-VEH	HFD-VEH vs. HFD-LA
Hypothalamic regions							
PVN	111.50 ± 11.84	149.50 ± 11.35	108.20 ± 8.04	29	H_3,29_ = 10.0, **p = 0.018**		
					Z = − 0.9, p = 0.35	Z = − 2.8, **p = 0.005**	Z = 2.2, **p = 0.028**
PLH	116.20 ± 11.71	140.90 ± 8.01	105.80 ± 7.68	29	H_3,29_ = 11.2, **p = 0.01**		
					Z = − 1.4, p = 0.16	Z = − 2.9, **p = 0.003**	Z = 2.6, **p = 0.010**
VMH	103.10 ± 8.09	130.70 ± 10.45	114.50 ± 9.26	29	H_3,29_ = 5.9, p = 0.12		
LH	116.00 ± 8.89	131.00 ± 13.61	106.80 ± 9.53	29	H_3,29_ = 2.7, p = 0.45		
STN	152.50 ± 3.04	159.80 ± 24.60	122.60 ± 10.72	29	H_3,29_ = 10.0, **p = 0.018**		
					Z = − 3.2, **p = 0.001**	Z = − 1.8, p = 0.074	Z = 0.9, p = 0.37
PSTN	161.10 ± 7.98	158.60 ± 17.60	109.50 ± 9.21	27	H_3,27_ = 16.0, **p = 0.001**		
					Z = − 3.1, **p = 0.002**	Z = − 2.8, **p = 0.006**	Z = 1.9, p = 0.06
Cortical areas							
PL	110.20 ± 9.52	140.00 ± 9.47	99.83 ± 6.36	30	H_3,29_ = 7.9, **p = 0.047**		
					Z = − 0.6, p = 0.56	Z = − 2.5, **p = 0.012**	Z = 2.3, **p = 0.020**
IL	104.30 ± 7.97	140.90 ± 12.30	104.20 ± 6.64	30	H_3,29_ = 6.4, p = 0.09		
OFC	95.65 ± 7.86	143.01 ± 3.63	99.55 ± 7.55	30	H_3,29_ = 17.4, **p = 0.000**		
					Z = 0.34, p = 0.73	Z = − 3.4, **p = 0.001**	Z = 3.1, **p = 0.002**
ACC	92.29 ± 7.59	136.10 ± 12.56	99.93 ± 6.43	28	H_3,28_ = 8.4, **p = 0.038**		
					Z = 0.8, p = 0.44	Z = − 2.2, **p = 0.027**	Z = 2.1, **p = 0.039**
aIC	115.90 ± 8.44	141.40 ± 6.41	109.50 ± 10.17	29	H_3,29_ = 11.9, **p = 0.007**		
					Z = − 1.9, p = 0.06	Z = − 3.2, **p = 0.002**	Z = 2.2, **p = 0.028**
Subcortical regions							
DMS	90.00 ± 5.13	151.50 ± 16.59	105.30 ± 3.26	28	H_3,28_ = 12.5, **p = 0.005**		
					Z = 1.4, p = 0.16	Z = − 2.5, **p = 0.012**	Z = 1.9, p = 0.05
DLS	104.30 ± 3.88	159.70 ± 13.65	107.90 ± 7.30	28	H_3,28_ = 14.1, **p = 0.002**		
					Z = − 0.8, p = 0.44	Z = − 3.1, **p = 0.002**	Z = 2.5, **p = 0.014**
dBNST	89.15 ± 6.57	153.20 ± 10.71	103.90 ± 5.62	29	H_3,29_ = 17.5, **p = 0.000**		
					Z = 0.81, p = 0.42	Z = − 3.3, **p = 0.001**	Z = 3.0, **p = 0.003**
dHipp	90.07 ± 5.24	139.80 ± 17.11	100.20 ± 8.18	29	H_3,29_ = 5.6, p = 0.14		
LHb	121.70 ± 8.34	142.50 ± 13.38	104.40 ± 8.75	29	H_3,29_ = 9.2, **p = 0.026**		
					Z = − 1.7, p = 0.08	Z = − 2.4, **p = 0.016**	Z = 2.2, **p = 0.028**
BLA	122.10 ± 6.34	144.10 ± 13.19	103.40 ± 9.86	29	H_3,29_ = 8.5, **p = 0.036**		
					Z = − 1.9, p = 0.06	Z = − 2.2, **p = 0.027**	Z = 2.2, **p = 0.028**
CeA	138.80 ± 10.91	147.70 ± 17.72	99.42 ± 14.81	29	H_3,29_ = 8.9, **p = 0.03**		
					Z = − 2.4, **p = 0.015**	Z = − 2.0, **p = 0.046**	Z = 1.8, p = 0.07
VPM	121.90 ± 3.07	201.70 ± 38.52	142.10 ± 18.63	29		H_3,29_ = 6.5, p = 0.09	
VPPC	115.80 ± 7.62	140.20 ± 16.46	86.53 ± 6.19	29	H_3,29_ = 9.3, **p = 0.025**		
					Z = − 1.6, p = 0.11	Z = − 1.9, p = 0.059	Z = 2.5, **p = 0.014**

Significant values are in bold.

Food regimen: standard diet—ST; high-fat diet—HFD. Treatment: vehicle—VEH; linoleic acid supplementation—LA. Data are means ± SEM. Paraventricular nucleus of the hypothalamus—PVN, peduncular part of the lateral hypothalamus –PLH, ventromedial hypothalamus—VMH, lateral hypothalamus—LH, subthalamic nucleus of the hypothalamus—STN, parasubthalamic nucleus of the hypothalamus—PSTN, prelimbic cortex—PL, infralimbic cortex—IL, orbitofrontal cortex—OFC, anterior cingulate cortex—ACC, anterior insular cortex—aIC, dorsomedial striatum—DMS, dorsolateral striatum—DLS, dorsal region of the bed nucleus of the stria terminalis—dBNST, dorsal hippocampus—dHipp, lateral habenula—LHb, basolateral amygdala—BLA, central amygdala—CeA, ventral posteromedial thalamic nucleus—VPM, and parvicellular part of the ventral posterior thalamic nucleus—VPPC. n: sample size.

**Figure 3:**
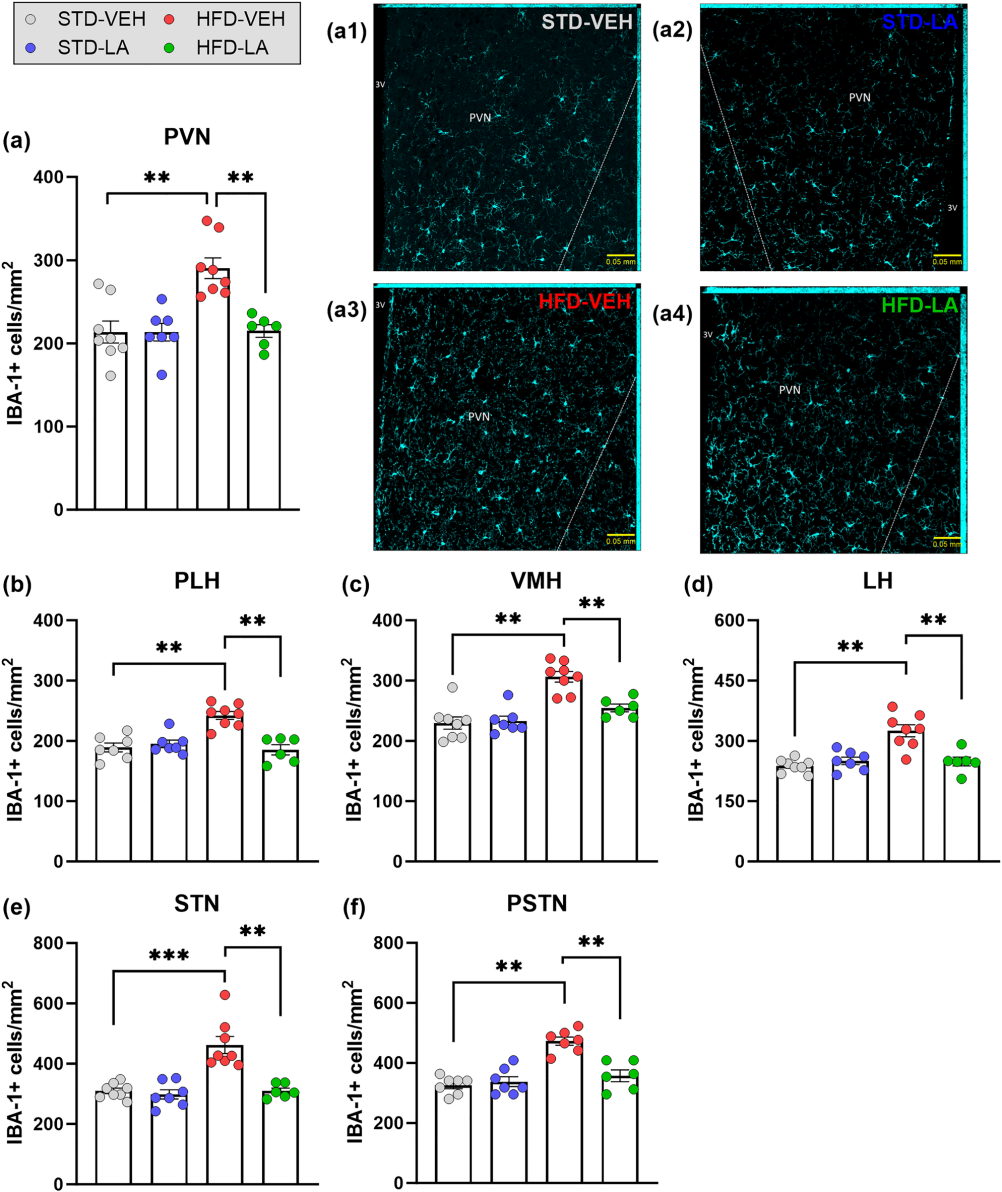
Hypothalamic microglial density assessed through IBA-1 immunofluorescence staining: evaluation of 5 weeks of linoleic acid (LA) supplementation on sustained high-fat diet (HFD)-induced neuroinflammation. A significant increase in IBA-1 positive (IBA-1+) cells in obese animals (HFD-VEH) compared to control mice (STD-VEH) indicates microgliosis consequent to the sustained fat-rich diet in hypothalamic areas: paraventricular nucleus (PVN) (**a**), peduncular part of the lateral hypothalamus (PLH) (**b**), ventromedial hypothalamus (VMH) (**c**), lateral hypothalamus (LH) (**d**), subthalamic nucleus (STN) (**e**) and parasubthalamic nucleus (PSTN) (**f**). Linoleic acid supplementation significantly reduced microglial density in all the hypothalamic regions evaluated. Illustrative photomicrographs of PVN IBA-1+ cells for the experimental conditions STD-VEH (**a1**), STD-LA (**a2**), HFD-VEH (**a3**) and HFD-LA (**a4**), taken with a ZEISS Axio Imager Z2 microscope, equipped with ApoTome.2 and a Camera ORCA-Flash4.OLT (*Zeiss*, Germany), and processed to get maximal orthogonal intensity projections. Values are mean ± SEM (n = 6–8 per group). **, p < 0.01; ***, p < 0.001.

**Figure 4:**
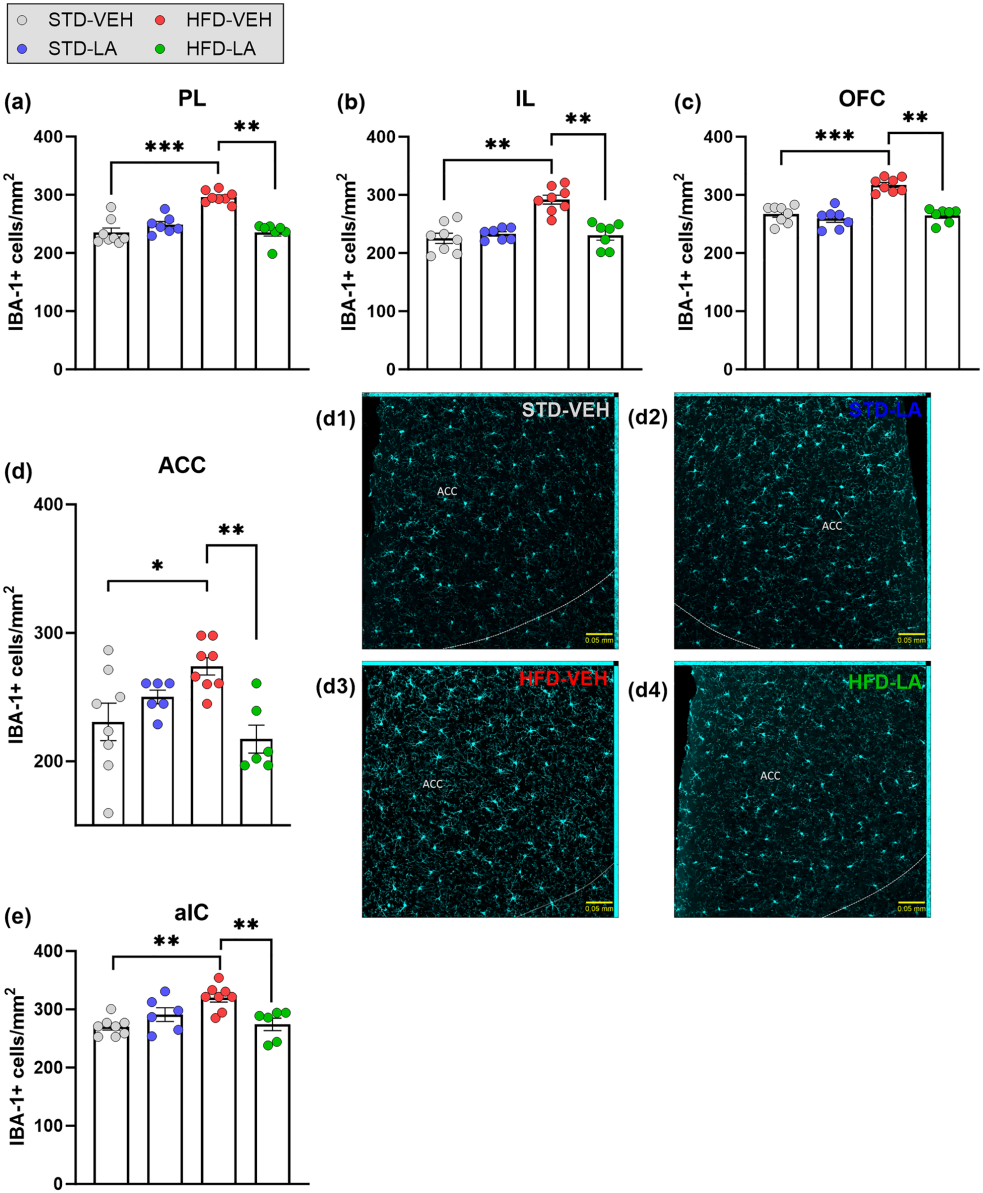
Cortical microglial density assessed by IBA-1 immunofluorescence staining: evaluation of 5 weeks of linoleic acid (LA) supplementation on sustained high-fat diet (HFD)-induced neuroinflammation. The figure shows a significant increase in IBA-1 positive (IBA-1+) cells in obese mice (HFD-VEH) compared to non-obese animals (STD-VEH) in all cortical regions evaluated: prelimbic cortex (PL) (**a**), infralimbic cortex (IL) (**b**), orbitofrontal cortex (OFC) (**c**), anterior cingulate cortex (ACC) (**d**) and anterior insular cortex (aIC) (**e**). Without exception, LA supplementation for 5 weeks significantly reduced microglial density in the investigated cortical structures (**a**–**e**). Illustrative photomicrographs of ACC IBA-1+ cells for the experimental conditions STD-VEH (**d1**), STD-LA (**d2**), HFD-VEH (**d3**) and HFD-LA (**d4**), taken with a ZEISS Axio Imager Z2 microscope, equipped with ApoTome.2 and a Camera ORCA-Flash4.OLT (*Zeiss*, Germany), and processed to get maximal orthogonal intensity projections. Values are mean ± SEM (n = 6–8 per group). *, p < 0.05; **, p < 0.01; ***, p < 0.001.

**Figure 5:**
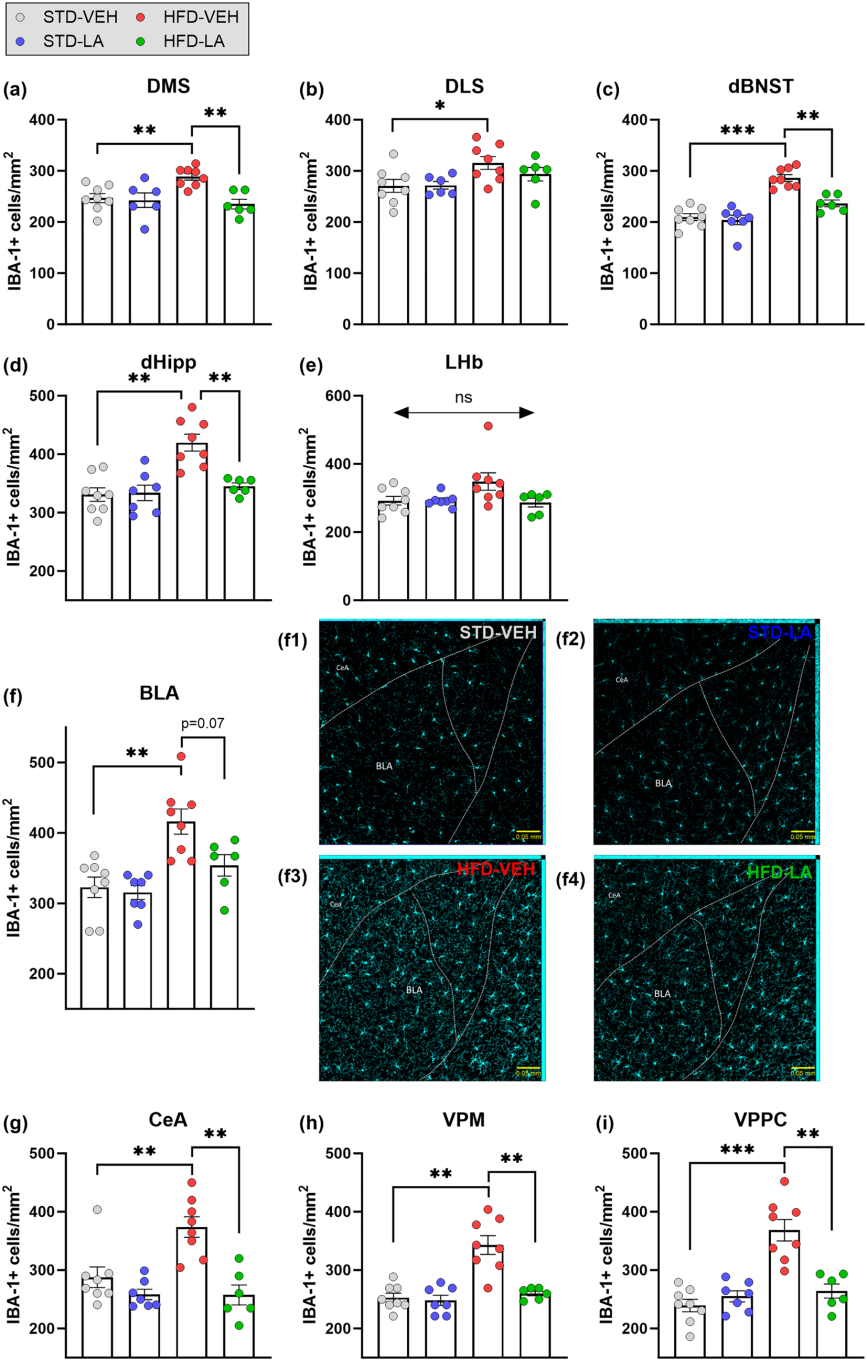
Subcortical microglial density assessed by IBA-1 immunofluorescence staining: evaluation of 5 weeks of linoleic acid (LA) supplementation on sustained high-fat diet (HFD)-induced neuroinflammation. Compared to animals fed a standard diet (STD-VEH), the density of IBA-1 positive (IBA-1+) cells was significantly increased in mice after a prolonged HFD (HFD-VEH) at subcortical level: dorsomedial striatum (DMS) (**a**), dorsolateral striatum (DLS) (**b**), dorsal part of the bed nucleus of the stria terminalis (dBNST) (**c**), dorsal hippocampus (dHipp) (**d**)**,** basolateral (BLA) (**f**) and central (CeA) (**g**) amygdala, ventral posteromedial thalamic nucleus (VPM) (**h**), and parvicellular part of the ventral posterior thalamic nucleus (VPPC) (**i**). No effect of the dietary regimen on microglial density was observed in the lateral habenula (LHb) (**e**). At the subcortical level, 5 weeks of LA supplementation significantly reduced microglial-related neuroinflammation in various structures (**a**, **c**, **d**, **g**–**i**), and a tendency to decrease was also revealed (**f**). Illustrative photomicrographs of BLA IBA-1+ cells for the experimental conditions STD-VEH (**f1**), STD-LA (**f2**), HFD-VEH (**f3**) and HFD-LA (**f4**), taken with a ZEISS Axio Imager Z2 microscope, equipped with ApoTome.2 and a Camera ORCA-Flash4.OLT (*Zeiss*, Germany), and processed to get maximal orthogonal intensity projections. Values are mean ± SEM (n = 6–8 per group). *, p < 0.05; **, p < 0.01; ***, p < 0.001; ns, not significant.

The neuroinflammatory effect of the HFD was first evaluated in the hypothalamic brain structures. In terms of cellular density, a main effect of HFD was evidenced in all the regions evaluated, thus confirming a general microgliosis at the local level (Fig. 3). In regard to IBA-1-related fluorescence intensity, a main effect of HFD was evidenced in the PVN, the PLH and the PSTN, as well as a tendency to increase in the STN. However, no differential IBA-1 expression between experimental conditions was found in the VMH, nor in the LH (Supplementary Fig. S1). Subsequently, the effect of the regimen was evaluated in the cortical structures. Microglial density of HFD individuals was significantly increased in all the cortical regions evaluated (Fig. 4). The comparison between fluorescence intensities also showed a global significant increase in IBA-1 expression at the cortical level, with the only exception being the IL (Supplementary Fig. S2). Finally, a significant increase of microglial density consequence of the HFD was evidenced in all the subcortical brain regions studied, with the only exception of the LHb (Fig. 5). IBA-1 relative expression was globally significantly increased at the subcortical level, with the exception of the VPPC, where only a tendency was detected, and of the dHipp and VPM, where no main effect of the regimen was evidenced (Supplementary Fig. S3).

The evaluation of the anti-inflammatory properties of LA supplementation in obese mice revealed an overall decrease in microglial density and IBA-1 expression. Hypothalamic microglial density significantly decreased after LA supplementation in all the structures evaluated (Fig. 3). Compared to the HFD-VEH group, HFD-LA animals showed reduced hypothalamic IBA-1-related fluorescence in the PVN and PLH, and a tendency in the PSTN. However, no significant effect of LA supplementation on IBA-1-related fluorescence was observed in the STN (no main effect observed in the VMH and LH) (Supplementary Fig. S1). Five weeks of LA supplementation also reduced obese mice’s microglial density and IBA-1 expression at the cortical level, with the only exceptions being the aIC (Fig. 4) and the IL (no main effect revealed, Supplementary Fig. S2), respectively. Concerning the subcortical level, a global beneficial effect of LA administration was observed on HFD-induced microgliosis, though only a tendency was detected in the BLA, and no significant effect in the DLS (no main effect in the LHb) (Fig. 5). A significant decrease of IBA-1-related fluorescence intensity was observed in the DLS, dBNST, BLA, LHb and VPPC, as well as a tendency in the DMS and CeA (no main effect in the dHipp and VPM) (Supplementary Fig. S3).

Concerning LA supplementation in non-obese animals, microglial density was found unaltered regardless of the region evaluated (Figs. 3, 4 and 5). Concerning IBA-1 expression, LA supplementation increased relative fluorescence intensity only in the CeA, PSTN and STN of STD-LA mice compared to control animals, and a tendency was observed in the aIC, LHb and BLA. No other effects were observed (Supplementary Figs. S1–S3).

### Morphometric evaluation of microglial reactivity

Microglial reactivity was further investigated through the morphological adaptations induced by HFD and LA supplementation. Microglial complexity in terms of cell ramification was evaluated in three brain structures, i.e. the PVN, the ACC and the BLA, respectively representing the hypothalamic, cortical and subcortical anatomical groups.

**Figure 6:**
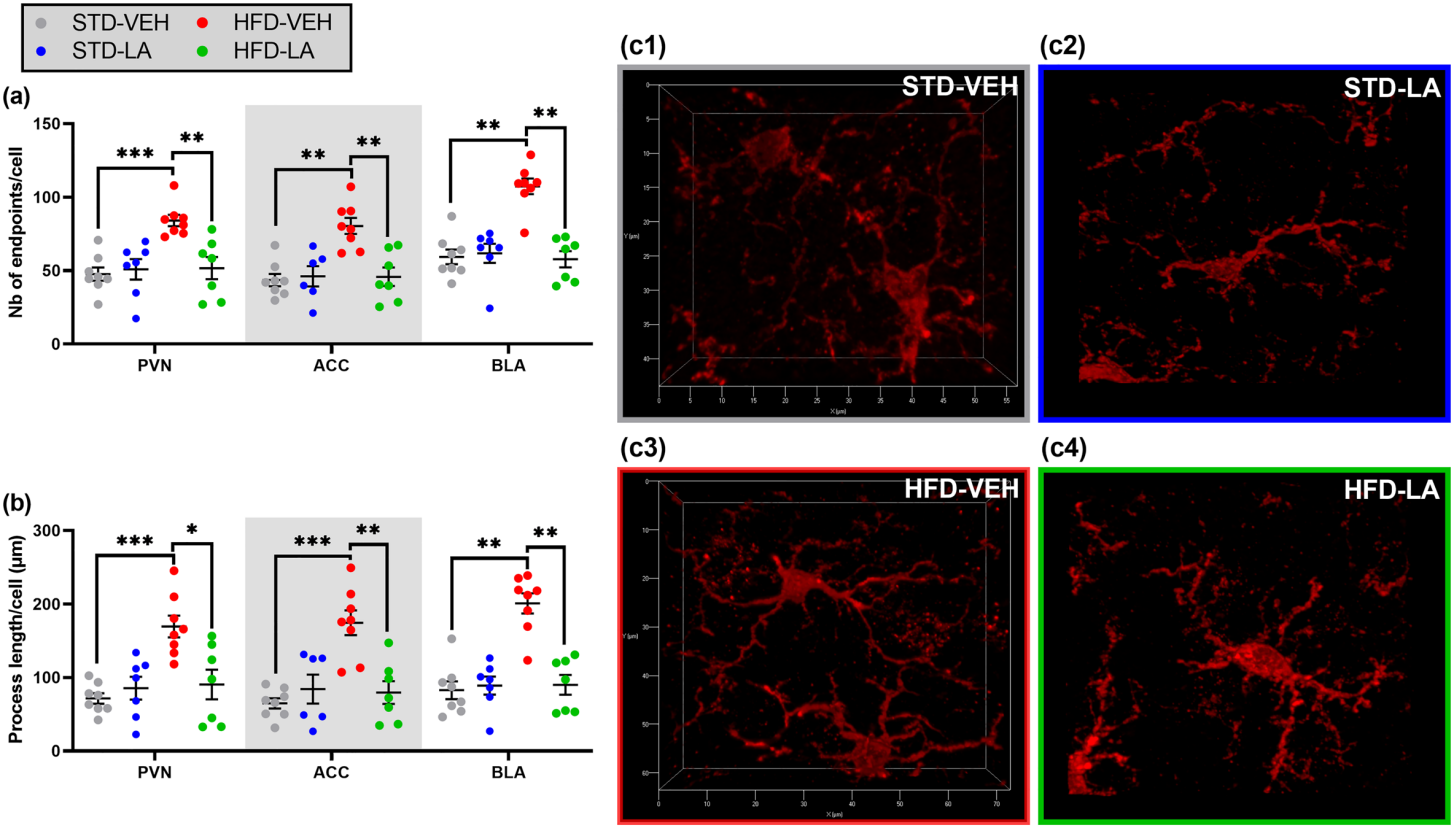
Morphometric study of microglial complexity: evaluation of 5 weeks of linoleic acid (LA) supplementation on sustained high-fat diet (HFD)-induced neuroinflammation. An increase of microglial complexity in terms of ramification (i.e. endpoints) (**a**) and perimeter of action (i.e. process length) (**b**) was found in mice under HFD (HFD-VEH) compared to control animals (STD-VEH) at hypothalamic (paraventricular nucleus—PVN), cortical (anterior cingulate cortex—ACC) and subcortical (basolateral amygdala—BLA) level. Linoleic acid supplementation reversed the microglial pathological phenotype induced by the dietary regimen (HFD-VEH vs. HFD-LA) in the regions investigated (**a**, **b**). No effect of LA supplementation on microglial morphology was observed in non-obese individuals (STD-VEH vs. STD-LA) (**a**, **b**). Illustrative 3D reconstructions of microglial cells for the experimental conditions STD-VEH (**c1**), STD-LA (**c2**), HFD-VEH (**c3**), and HFD-LA (**c4**), taken with a ZEISS Axio Imager Z2 microscope, equipped with ApoTome.2 and a Camera ORCA-Flash4.OLT (*Zeiss*, Germany). Values are mean ± SEM (n = 6–8 per group). *, p < 0.05; **, p < 0.01; ***, p < 0.001.

As illustrated in Fig. 6a, the HFD regimen significantly increased microglia’s ramification in the PVN (mean number of endpoints ± SEM, STD-VEH: 47.64 ± 4.54; HFD-VEH: 84.14 ± 3.88) [KW: H_3,30_ = 16.1, p = 0.001; *post-hoc* MWU, Z = − 3.4, p = 0.0008], the ACC (STD-VEH: 43.62 ± 4.16; HFD-VEH: 80.42 ± 5.42) [H_3,29_ = 13.8, p = 0.003; Z = − 3.2, p = 0.0016] and the BLA (STD-VEH: 59.43 ± 5.01; HFD-VEH: 107.38 ± 5.32) [H_3,30_ = 17.1, p = 0.000; Z = − 3.3, p = 0.0011]. Likewise, the process length of microglial cells was significantly increased in animals under HFD compared to their control counterparts in the three regions investigated (PVN, STD-VEH: 71.50 ± 7.08; HFD-VEH: 169.56 ± 14.77; ACC, STD-VEH: 64.85 ± 7.03; HFD-VEH: 174.54 ± 16.83; BLA, STD-VEH: 82.75 ± 12.08; HFD-VEH: 200.94 ± 13.66) [PVN: H_3,30_ = 14.3, p = 0.002; Z = − 3.4, p = 0.0008; ACC: H_3,29_ = 13.8, p = 0.003; Z = − 3.4, p = 0.0002; BLA: H_3,30_ = 16.2, p = 0.001; Z = − 3.3, p = 0.0011] (Fig. 6b).

The evaluation of LA supplementation on microglia’s morphological complexity revealed a significant effect of the treatment in obese mice, with HFD-LA individuals having a less ramified microglia (PVN: 51.67 ± 7.57; ACC: 45.84 ± 6.36; BLA: 57.75 ± 5.54) than non-treated individuals under HFD [PVN, Z = 2.9, p = 0.0038; ACC, Z = 2.8, p = 0.0055; BLA, Z = 3.2, p = 0.0012] (Fig. 6a). In addition, a decrease of the process length of microglial cells was observed in obese individuals treated with the LA solution (PVN: 90.65 ± 20.22; ACC: 79.60 ± 15.52; BLA: 90.05 ± 13.58) [PVN, Z = 2.4, p = 0.015; ACC, Z = 2.9, p = 0.0038; BLA, Z = 3.1, p = 0.0018] (Fig. 6b).

Regardless of the region investigated, LA supplementation in non-obese individuals did not affect microglia’s morphological complexity in terms of ramification (STD-LA, PVN: 50.91 ± 6.97; ACC: 46.14 ± 6.85; BLA: 61.78 ± 6.53) [PVN, Z = − 0.7, p = 0.49; ACC, Z = − 0.3, p = 0.80; BLA, Z = − 1.0, p = 0.30], nor of process length (STD-LA, PVN: 85.60 ± 15.43; ACC: 84.34 ± 19.68; BLA: 89.09 ± 12.16) [PVN, Z = − 0.9, p = 0.35; ACC, Z = − 0.3, p = 0.80; BLA, Z = − 0.8, p = 0.42] (Fig. 6a, b).

### Evaluation of microglial contribution to anxio-depressive-like behavior

Principal component analyses (PCAs) were used to explore the relationship between animals’ emotional behavior (LDB test and ST parameters, due to reported behavioral alteration), and microglial density and IBA-1 relative expression in hypothalamic, cortical and subcortical regions separately.

**Figure 7:**
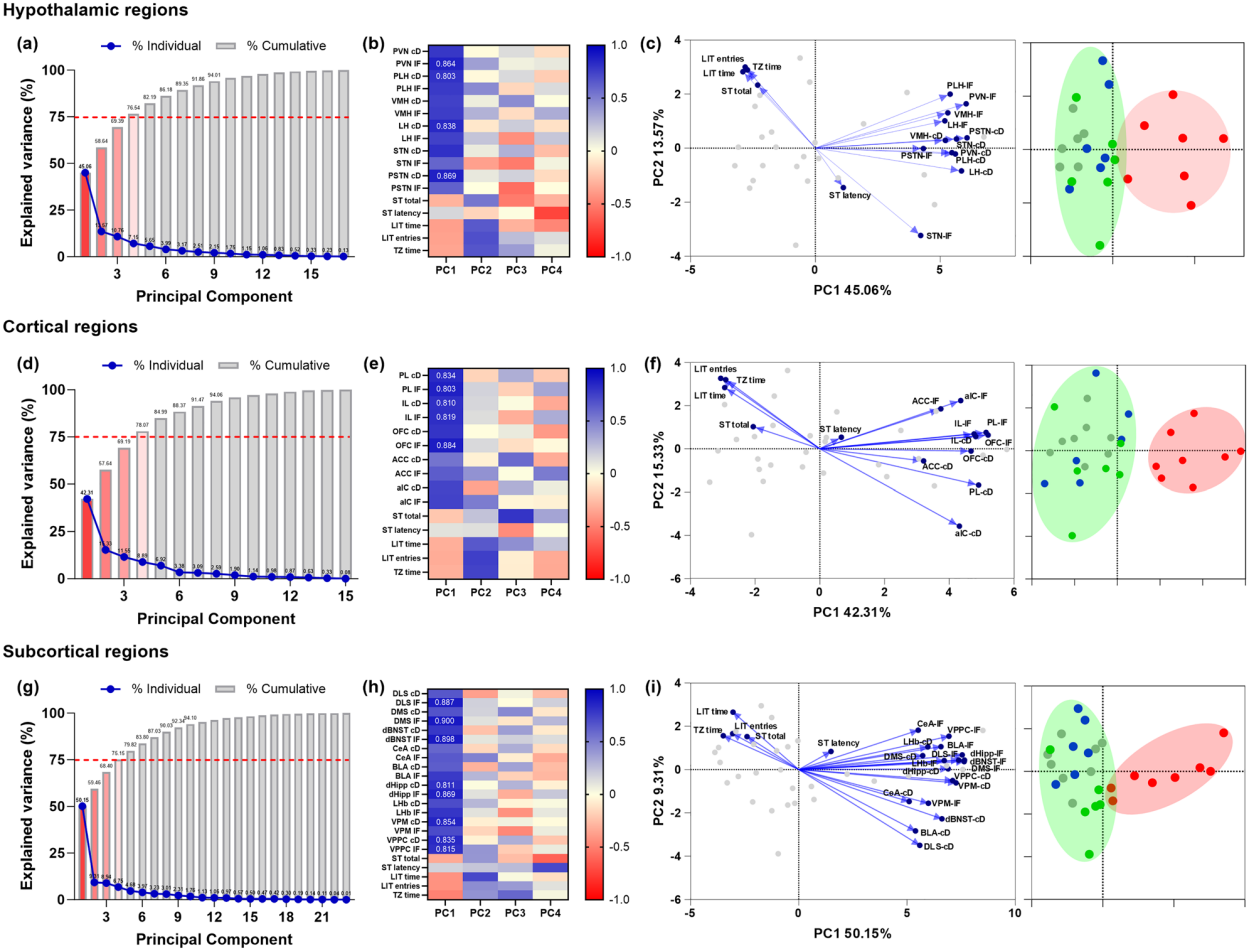
Relationship between behavioral scores in the light–dark box (LDB) test and the splash test (ST), and microglial-related neuroinflammation in the studied brain regions. From the overall results, the principal component analyses (PCAs) revealed that four principal components (PC1-4) explain 75% of the variance of the data sets (hypothalamic, **a**; cortical, **d**; and subcortical regions, **g**). Heat-maps of the loading values of PC1-4 show that microglial density and IBA-1 relative expression strongly correlate with PC1 for the three data sets (**b**, **e**, **h**), and that behavioral scores mostly correlate with PC2 in hypothalamic (**b**) and cortical regions (**e**). Only the time spent in the lit compartment during the LDB test correlates with PC2 at subcortical level (**h**). Biplots for each data set show IBA-1 relative expression predicts microglial density, and that ST and LDB behavioral scores are slightly predicted by the magnitude of microgliosis (**c**, **f**, **i**). Biplots illustrate two clusters of individuals that assemble in terms of microglial-related neuroinflammation. Thus, the pathological experimental group (HFD-VEH) shapes one of the clusters (**c**, **f, i**, red dots**)**, and the second cluster comprises non-obese or obese animals where neuroinflammation had been rescued (**c**, **f**, **i**; respectively: STD-VEH, grey dots; STD-LA, bleu dots; HFD-LA, green dots). Entries in the lit compartment –LIT entries; time spent in the lit compartment, –LIT time; time in the transition zone, –TZ time; latency to first grooming event, –ST latency; total time grooming, –ST total.

Based on the PCA results, 4 principal components (PC1-4) explain more than 75% of the total variance of each data set’s metrics. As illustrated in Fig. 7, the first two PCs (PC1 and PC2) explain 58.64% of the total variance for the hypothalamic regions (Fig. 7a), 57.64% for the cortical regions (Fig. 7d) and 59.46% for the subcortical regions (Fig. 7g). The loading values (i.e. correlation values from data vs. eigenvectors) of PC1-4 representing the majority of the variation of the data sets are plotted as *heat-maps* for better interpretation of the contribution of single variables within single PCs. As a general pattern, microglial density and IBA-1 relative expression strongly correlate with PC1 in all hypothalamic (Fig. 7b), cortical (Fig. 7e) and subcortical regions (Fig. 7h), with PC1 increasing as microglial density and IBA-1 relative expression increase. Concerning the behavioral parameters, they mostly correlate with PC2 in hypothalamic and cortical regions, with the exception being the ST latency. In the subcortical data set, only the time spent in the lit compartment during the LDB test correlates with PC2. According to the PCA results, the observed increase in IBA-1 expression consequence of feeding the animals a HFD predicts local microgliosis in terms of cellular density. However, as illustrated in the PCA biplots, behavioral scores in the ST and the LDB test are only modestly predicted by the severity of microgliosis, irrespective of the anatomical location (Fig. 7c, f, i). The PCA scores of samples at the right of the PCA biplots (Fig. 7c, f, i) illustrate the similarity of the experimental groups in terms of the parameters evaluated, and show two main clusters. These clusters suggest that microglial reactivity might differently account for emotional behavior characterizing (1) HFD-induced obese mice and (2) non-obese animals (STD-VEH and STD-LA) or obese animals where neuroinflammation had been rescued (HFD-LA). Complementary correlation analyses suggest that five anatomical areas, i.e. DMS, dHipp, LHb, VPM, and STN—might serve as main actors explaining a relationship between microgliosis and the behavior observed in the LDB test (Supplementary Fig. S4). Mice with higher IBA-1 fluorescence intensities in these brain regions consistently spent shorter periods in the transition zone compared to those with lower IBA-1 intensities [DMS, r = − 0.426, p = 0.024; dHipp, r = − 0.594, p = 0.0007; LHb, r = − 0.597, p = 0.049; VPM, r = − 0.695, p < 0.0001; STN, r = − 0.599, p = 0.0006] and visited the lit compartment less often [dHipp, r = − 0.373, p = 0.047; LHb, r = − 0.341, p = 0.07; VPM, r = − 0.436, p = 0.018; STN, r = − 0.315, p = 0.10]. However, the variation in the data set explained by these linear models does not reach 50% for all the animals. Interestingly, if the HFD-VEH group is studied separately, the explained variance with these linear models (R^2^ values) increases considerably [HFD-VEH, time in transition: dHipp, r = − 0.759, p = 0.029; LHb, r = − 0.726, p = 0.041; VPM, r =− 0.894, p = 0.027; STN, r = − 0.758, p = 0.029; entries into lit comp.: DMS, r = − 0.563, p = 0.073; dHipp, r = − 0.815, p = 0.014; LHb, r = − 0.790, p = 0.020; VPM, r = − 0.906, p = 0.0019; STN, r = -0.787, p = 0.021], which is not the case for the other experimental groups [STD-VEH, STD-LA and HFD-LA, time in transition: DMS, r = − 0.045, p = 0.84; dHipp, r = − 0.360, p = 0.11; LHb, r = − 0.405, p = 0.07; VPM, r = − 0.545, p = 0.016; STN, r = − 0.398, p = 0.074; entries into lit comp.: DMS, r = − 0.338, p = 0.15; dHipp, r = − 0.055, p = 0.82; LHb, r = − 0.022, p = 0.92; VPM, r = − 0.169, p = 0.46; STN, r = − 0.042, p = 0.86]. These results therefore support a differential relationship between microgliosis and behavior for HFD-induced obese mice and for obese (HFD-LA) or non-obese animals (STD-VEH and STD-LA) in absence of microglial-related neuroinflammation.

## Discussion

The mechanisms underlying chronic neuroinflammation as a consequence of high fat consumption remain under-studied. Furthermore, excessive dietary fat intake and obesity enhance an individual’s vulnerability to emotional disturbances^6,10–12^. Our study shows that high-saturated-fat-induced neuroinflammation extends beyond the hypothalamus, significantly impacting cortical and subcortical brain regions. Neuroinflammation was observed concomitant to an anxio-depressive-like symptomatology in obese mice. Our work also confirms the anti-neuroinflammatory properties of LA supplementation and demonstrates its therapeutic potential at the chronic stage of neuroinflammation in a murine model of diet-induced obesity.

In line with previous studies^11,30–32^, we show that sustained consumption of a fatty diet leads to a significant increase of the body weight, which to a certain extent, is a consequence of an installed hyperphagia. Although LA did not affect diet-induced obesity, it might have impacted energy and glucose homeostasis and therefore overall animal’s metabolic health affecting, directly or indirectly, neuroinflammation and behavior. Since we know that our high-fat regimen leads to insulin resistance and peripheral inflammation, as show in our previous publication^30^, it would have been informative to include some physiological measurements like post-fasting glycemia in order to evaluate the contribution of LA supplementation at peripheral level. As expected, drinking intake was significantly reduced in obese individuals compared to non-obese animals, and therefore mean LA intake during the 5 weeks of treatment differed between the STD-LA and HFD-LA experimental groups. Future investigation should consider the effect of HFD on drinking intake and perhaps adapt the LA administration protocol, in order to address its contribution to inter-group differences.

The emotional disturbances observed here consequent to sustained high saturated fat consumption agree with several reports (e.g.,^10^). Compared to control mice, the grooming and lit compartment exploration times (in the ST and LDB, respectively) are significantly reduced in obese mice. However, no altered emotional behavior was observed in either the NSF task or NST. Therefore, the latency to first approach food could not sufficiently illustrate the motivational imbalance characterizing obese mice, and the HFD regimen did not induce compulsive-like behaviors.

Although beneficial effects of dietary supplementation on emotional disturbances have been reported^13,33^, neither anti-depressive nor anxiolytic effects of 5 weeks of LA supplementation were observed in our investigation. Interestingly, Demers et al.^13^ demonstrated that emotionality in HFD-induced obese mice was alleviated when the regimen was supplemented with polyunsaturated ω-3 fatty acids; we did not come to the same conclusion, though we used an equivalent treatment duration. This difference might be related to the use of a ω-6 fatty acid instead of a ω-3 fatty acid, but also to the administration protocol, which differed between studies (oral vs. gavage administration). Future complementary metabolic analyses might help us understand why 5 weeks of LA supplementation were not sufficient to improve the emotionality of obese mice. Increasing the LA treatment duration in a complementary study might also benefit the understanding of the discrepancies observed between microglial-related measurements and animals’ emotionality. It would also be interesting to explore whether the combination of different polyunsaturated fatty acid leads to beneficial synergetic effects on animals’ behavior.

Obesity-related neuroinflammation has been described in hypothalamic regions due to their key role in energy homeostasis and food intake regulation (for a review, see^34^). Interestingly, our study demonstrates that the inflammatory profile of obesity extends to the entire CNS. Microgliosis as a consequence of high-fat regimens occurs notably early: feeding a HFD for 1–4 days is sufficient to induce changes in microglia^35,36^. Nevertheless, literature alludes to the chronicity of neuroinflammation as a key feature for the emergence of emotional disturbances. We therefore assessed the anti-neuroinflammatory properties of LA, as well as its impact on obesity-induced emotionality^28,37^, while supplementing a high-saturated-fat diet. Five weeks of LA administration rescued overall cerebral microgliosis (HFD-VEH vs. HFD-LA) and switch the activation state of the microglial population (i.e. from hyper-ramified back to physiological-like morphology). Experimental HFDs have been shown to significantly reduce cerebral LA content (for instance^13^). While LA metabolites participate in the control of the peripheral inflammatory response, LA has been suggested to modulate microglial function by binding free fatty acid receptors 1 and 4 (FFAR1 and FFAR4), and fatty acid binding proteins (FABPs) on their surface^38^. Hence, exogenous LA supplementation might have reversed the effect of HFD on brain LA content, and therefore rescued this anti-inflammatory pathway. Concerning our results, since we showed that LA exerts overall anti-inflammatory effects in the brain, an improvement in animals’ emotionality was expected. Unfortunately, the behavioral phenotype of obese mice in the ST and the LDB test did not benefit from LA supplementation. The lack of behavioral effect might be attributed to the duration of the treatment.

Another noteworthy observation is the increased IBA-1 expression in the STN, PSTN, and CeA of control mice after LA supplementation. These regions participate in the regulation of food intake, and the PSTN in particular is involved in refraining from feeding^39,40^. Thus, the isolated LA effect suggests that these regions might be especially sensitive to the treatment and therefore, to dietary variations of fat intake. In addition to an under-characterized effect of central inflammation in terms of regional specificity and time course, the neural-glial dynamics of HFD-induced hypothalamic inflammation are not yet clearly understood. We hypothesize that STN and PSTN act as highly sensitive sensors of fat variations within the hypothalamus, potentially through CD36 fat-receptor expression, and enhance the inflammatory response through different synergistic effects of neural-originated and glia-originated inflammation. In fact, hypothalamic and extra-hypothalamic brain regions express CD36, allowing long-chain fatty acids to cross the blood–brain barrier and influence neuronal activity^41,42^. Further investigation on the effects of LA supplementation on other glial populations, and in particular, on astrocytes, which along with microglia are major actors of the neuroinflammatory response, would certainly help understanding the underlying processes.

In our exploration of the relationship between emotionality and the severity of neuroinflammation, we confirmed that an increase of IBA-1 expression in our model of diet-induced obesity predicted microgliosis in terms of cellular density. Although the PCAs results show that microgliosis is a poor predictor of animals’ behavior in the LDB and the ST, individuals’ PC scores aggregated into two clusters that differ in terms of the severity of microgliosis. Additionally, while linear models could not explain the overall variation of the data sets, they better predicted microgliosis through behavioral scores in untreated obese animals (see Supplementary data). These observations therefore suggest that the identification of microglial heterogeneity and signature is crucial in order to study the relationship between neuroinflammation and emotional imbalance.

Combined, our findings support the use of dietary LA supplementation as a therapeutic tool for alleviating neuroinflammation associated with overconsumption of fatty food and obesity. Our results open new vistas in the management of obesity by the supplementation of linoleic acid, a long-chain fatty acid, in dietary interventions.

## Methods

### Animals

Forty 8-week-old male C57BL/6JRj mice (*Janvier Labs*, Saint-Berthevin, France) were used in this study. Animals were group-housed and maintained under a controlled environment (12-h light/dark cycle from 7 a.m.; temperature: 22 ± 2 °C; humidity: 55 ± 10%). They had ad libitum access to food (standard diet—STD, or high-fat diet—HFD; see “Dietary regimens”) and drinking water, vehicle or LA supplementation (see “Treatment”). All procedures were conducted in accordance with the Guide for the Care and Use of Laboratory Animals (NIH), the Animal Research: Reporting of In Vivo Experiments (ARRIVE) guidelines, and the European Union regulations on animal research (Directive 2010/63/EU). They were approved by the University of Franche-Comté Animal Care and Use Committee (CEBEA-58).

### Dietary regimens

Animals were randomly divided into two groups of 20 individuals each and were fed either an STD (KlibaNafag 3430PMS10, *Serlab*, CH-4303 Kaiserau, Germany) or a HFD (A03 food powder, *SAFE*, Augy, France; cholesterol C8667, *Sigma-Aldrich*, France; palm oil, *Vigean*, France) ad libitum as previously described^30^ for 17 weeks. See diet composition details in Supplementary Table S1.

### Treatment

After 12 weeks, mice were randomly divided as follows: half the animals in each group were treated with vehicle (VEH; 0.3% w/v, Arabic gum solution, in water; G9752, *Sigma-Aldrich*, France; STD-VEH, n = 10; HFD-VEH, n = 10), and the remainder were treated with a LA solution (0.2% LA, w/v, in VEH; L1376, *Sigma-Aldrich*, France; STD-LA, n = 10; HFD-LA, n = 10). The Arabic gum, which is an edible mixture of polysaccharides and glycoproteins, was used here as suspending agent since LA is immiscible in water. The LA dose was chosen based on previous studies^30^. Solutions were prepared three times a week in order to minimize LA oxidation, and the beverage intake was regularly monitored.

### Behavioral characterization

The behavioral tests started 17 weeks after the onset of the dietary regimens and after 5 weeks of VEH/LA administration. They were conducted during the light/inactive phase of the circadian cycle.

#### Nestlet shredding test (NST)

To evaluate repetitive, compulsive-like behavior, mice were tested in the NST, which can robustly measure the effects of different treatments on their natural and spontaneous nestlet shredding behaviour. Mice were individually placed into a cage with litter and a pre-weighed nestlet, and left undisturbed for 30 min. After the test completion, mice were replaced in their home cages and the remaining intact nestlet material was removed, dried overnight and weighed. This measure was used to calculate the percentage of the total nestlet that had been shredded, which served as an index of compulsive-like behavior^43,44^.

#### Splash test (ST)

Depressive-like behavior was investigated by leveraging rodents’ inherent drive to groom themselves during the ST. Briefly, after spraying the dorsal surface of its body with a 15% sucrose solution, each animal was individually placed for 5 min in an individual home cage with litter. A prolonged latency to starting the first grooming period and an increased total time of grooming were considered as indicative markers for a depressive-like phenotype, in accordance with the literature^45^.

#### Novelty-suppressed feeding (NSF) task

In rodents, the measure of hyponeophagia (i.e., the inhibition of feeding produced by a novel environment) in the NSF task informs about emotional alterations by appealing to the appetitive component of incentive motivation^46^. The NSF task has been proved sensitive to pharmacological treatments targeting anxio-depressive symptomatology^47,48^. Following a 12-h period of food deprivation, animals were individually placed in an opaque cylindrical open-field apparatus (height: 60 cm; diameter: 47 cm), in the center of which they could reach two grain-based pellets located on a white piece of filter paper (diameter: 10 cm). Prolonged latencies until their first approach towards the food served as a quantitative indicator of anxio-depressive-like behavior. The test lasted for a maximum of 10 min (adapted from^49^).

#### Light–dark box (LDB) test

In the LDB test, anxious-like behavior was assessed by subjecting animals to a choice paradigm involving an internal conflict between remaining in a dimly lit “safe” compartment (width: 40 cm; high: 14 cm; 20–30 lx) and venturing into a brightly lit “novel” one (width: 40; high: 26; 120–130 lx). The test duration was set at 10 min, during which the animal’s behavior was continuously monitored and recorded using an automatic videotracking system (*Viewpoint Behavior Technology*, Lyon, France). The behavioral metrics evaluated were the duration and frequency of entries into the lit compartment, the time spent in the transition zone, and the total distance traveled within the testing arena. An entry was defined as the placement of all four of the animal’s legs into either compartment. Otherwise, the animal was considered to be in the transition zone (adapted from^31^). A shorter exposure to the brightly lit compartment compared to the dimly lit compartment was considered as indicative of anxious-like behavior.

### Animal sacrifice and tissue sampling

Twenty-four hours after the behavioral assessment, animals were euthanized with an intraperitoneal injection of pentobarbital (55 mg/kg, Exagon®, *Med’Vet*, France) for neuroinflammatory evaluation through ionized calcium-binding adapter molecule 1 (IBA-1) immunofluorescence staining. The procedures were similar to those we previously described^50,51^. After, mice were transcardially perfused with 0.9% NaCl, followed by 4% paraformaldehyde fixative (PFA, *Roth®*, Karlsruhe, Germany) dissolved in 0.1 M phosphate buffer (PB, pH 7.4). Extracted brains were postfixed overnight at 4 °C in the same fixative and then cryoprotected for 24 h at 4 °C by immersion in a 15% sucrose solution (D(+)- Saccharose, *Roth®*, Karlsruhe, Germany) in a 0.1 M PB. Finally, the brains were rapidly frozen via immersion in isopentane (2-methylbutane, *Roth®*, Karlsruhe, Germany) and sliced into serial coronal 30-µm-thick sections.

### Immunofluorescence

Between six and eight individuals of each experimental group were randomly selected for assessing microglial reactivity in a selection of hypothalamic, cortical, and subcortical brain regions, based on their direct or indirect involvement in the homeostatic and/or hedonic regulation of food intake and anxio-depressive-like symptomatology: prelimbic (PL), infralimbic (IL), and orbitofrontal (OFC) cortices at 1.94–1.70 mm anterior to Bregma (aB); anterior cingulate cortex (ACC), dorsolateral (DLS) and dorsomedial (DMS) striatum at 1.10–0.98 mm aB; anterior insular cortex (aIC), paraventricular nucleus of the hypothalamus (PVN), and dorsal region of the bed nucleus of the stria terminalis (dBNST) at 0.26–0.02 mm aB; amygdala (central—CeA and basolateral—BLA) and peduncular part of the lateral hypothalamus (PLH) at 1.06–1.34 mm posterior to Bregma (pB); dorsal hippocampus (dHipp), lateral habenula (LHb), and ventromedial hypothalamus (VMH) at 1.70–1.94 mm pB; ventral posteromedial thalamic nucleus (VPM), parvicellular part of the ventral posterior thalamic nucleus (VPPC), lateral hypothalamus (LH), subthalamic nucleus (STN), and parasubthalamic nucleus (PSTN) at 2.06–2.30 mm pB, according to Franklin and Praxinos^52^. Floating sections were incubated with the primary antibody (1:2000; ab178846, rabbit anti-IBA1, *Abcam*) for 24 h at 4 °C, and then with the secondary antibody (1:1000; A10520, goat anti-rabbit IgG Cyanine 3, *Invitrogen*) for 2 h at room temperature. Finally, sections were mounted on gelatin-coated slides and cover-slipped with mounting media (40% PB, 60% glycerol; *Roth®*, Karlsruhe, Germany). Photomicrographs of brain structures for analysis were acquired using a 10 × objective of an Olympus microscope Bx51 equipped with an Olympus DP50 camera. *ImageJ* software was used to quantify microglial density (number of IBA-1 positive cells per given surface -IBA-1^+^/mm^2^) and IBA-1-related fluorescence (individual fluorescence intensities were related to the mean fluorescence intensity of the STD-VEH control group, and presented as percentages -%). See Table 1 for detailed final sample sizes.

### Data and statistical analyses

The results are presented as means ± SEM. The statistical analyses were conducted using STATISTICA 10 (*Statsoft*, Palo Alto, United States). Figures were designed using GraphPad Prism version 10.2.0 for Windows (GraphPad Software, Boston, Massachusetts USA, www.graphpad.com). Assumptions for parametric analysis were verified using Shapiro–Wilk and Levene’s tests to respectively study the data sets’ normality of distribution and homogeneity of variance. The progression of body weight, food intake and liquid intake was evaluated with a repeated-measures ANOVA (RMA) design, including two (STD and HFD) or four (STD-VEH, STD-LA, HFD-VEH, and HFD-LA) groups (between-subject factor: regimen or treatment) and 12 or five measurements (within-subject factor: time). When the data sets did not meet assumptions for parametric analysis (behavioral, cellular density, immunofluorescence scores and morphometric scores), Kruskal–Wallis (KW) and Mann–Whitney U (MWU) tests were used. Gehan–Breslow Wilcoxon (GBW) tests were used to compare NSF score curves. Morphometric analyses aiming at evaluating microglia’s morphological complexity in terms of ramification (i.e. endpoints) and perimeter of action (i.e. process length) were performed using *ImageJ* Fiji software (National Institute of Health, Bethesda, Maryland, USA, https://imagej.net/ij/^53^) as reported by Young and Morrison^54^. Principal component analyses (PCA) were performed in order to reduce redundancy of the information within the data sets and to maximize explanatory variance across measured parameters. The PCAs aimed to identify relevant components in the relationship between behavioral and microglia-related scores. For all analyses, the threshold for statistical significance was set at p < 0.05 and dependencies with p ≤ 0.1 were considered as trends.

### Supplementary Information

Supplementary Information.

## Data Availability

The datasets used and/or analysed during the current study available from the corresponding author on reasonable request.
