# Supplementary material for: Dietary linoleic acid supplementation protects against obesity-induced microglial reactivity in mice
Source: Sci Rep. 2024 Mar 19;14:6644. doi: 10.1038/s41598-024-56959-6 (PMC10951280; doi:10.1038/s41598-024-56959-6)
Supplement: Supplementary file 1 — Supplementary Information. [file 41598_2024_56959_MOESM1_ESM.pdf]

Supplementary Information

Dietary linoleic acid supplementation protects against obesity-induced microglial reactivity in mice

Authors

Lucas Jantzen[1], Stéphanie Dumontoy[1], Bahrie Ramadan[1], Christophe Houdayer[1], Emmanuel Haffen[1,2,3], Aziz Hichami[4], Naim Akhtar Khan[4], Vincent Van Waes[1#], Lidia Cabeza[1*#]

Affiliations

[1]Université de Franche-Comté, UMR INSERM 1322 LINC, F-25000 Besançon, France ; [2]Université de Franche-Comté, UMR INSERM 1322 LINC, service de Psychiatrie Clinique, CHU, F-25000 Besançon, France ; [3]Université de Franche-Comté, UMR INSERM 1322 LINC, CIC-INSERM 1431, CHU, F-25000 Besançon, France ; [4]Physiologie de la Nutrition & Toxicologie (NUTox), UMR UB/Institut Agro/ INSERM U1231, Lipides, Nutrition & Cancer, LABEX-LipStick, Université de Bourgogne, Dijon, France

Supplementary Figure S1: Hypothalamic IBA-1 relative expression assessed through immunofluorescence staining.

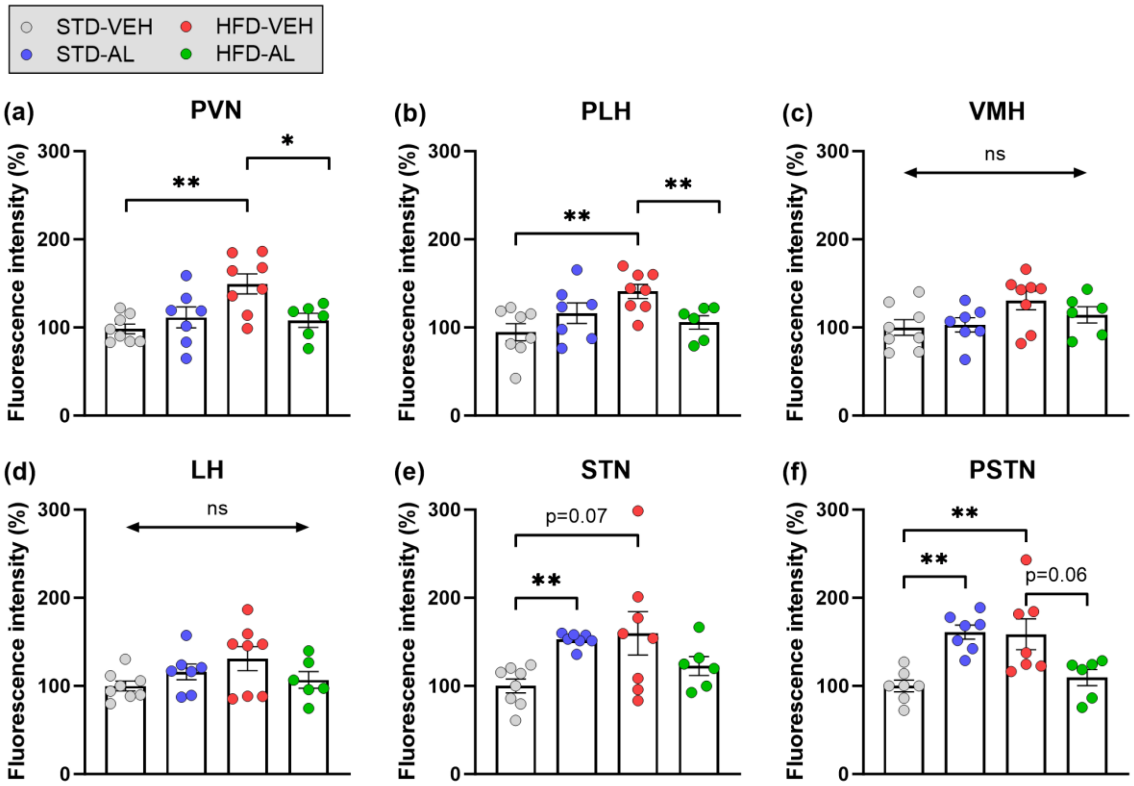

Supplementary Figure S2: Cortical IBA-1 relative expression assessed through immunofluorescence staining

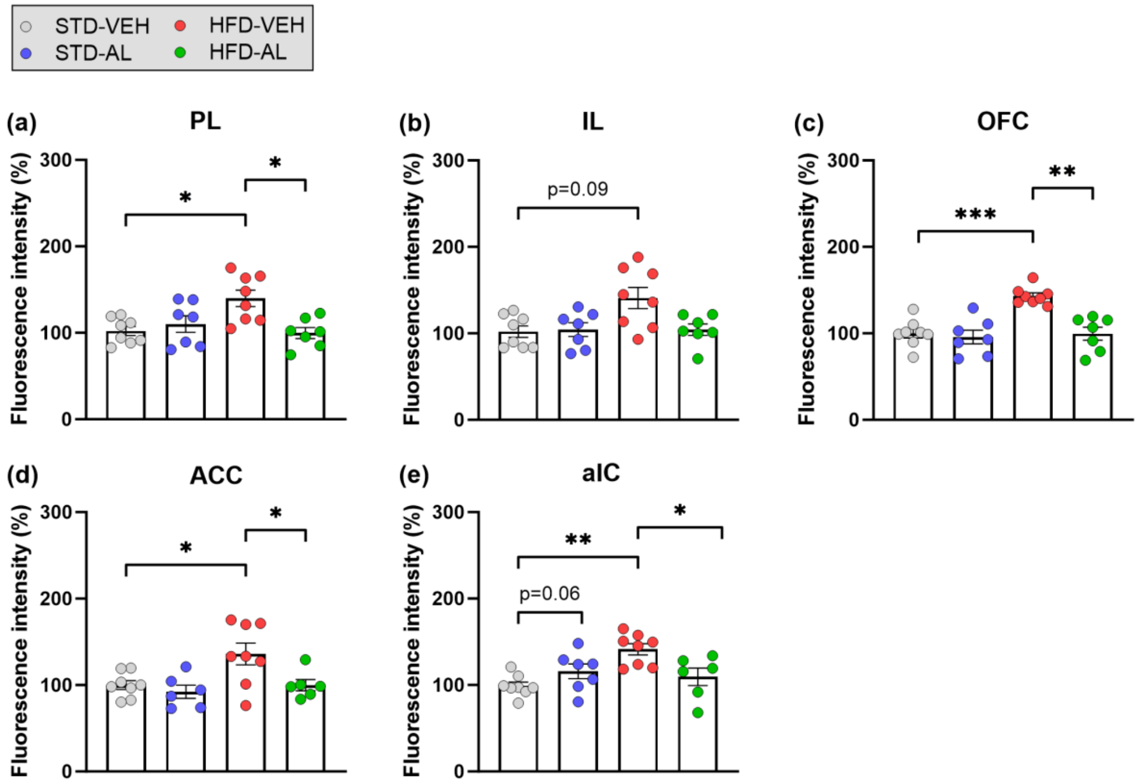

Supplementary Figure S3: Subcortical IBA-1 relative expression assessed through immunofluorescence staining.

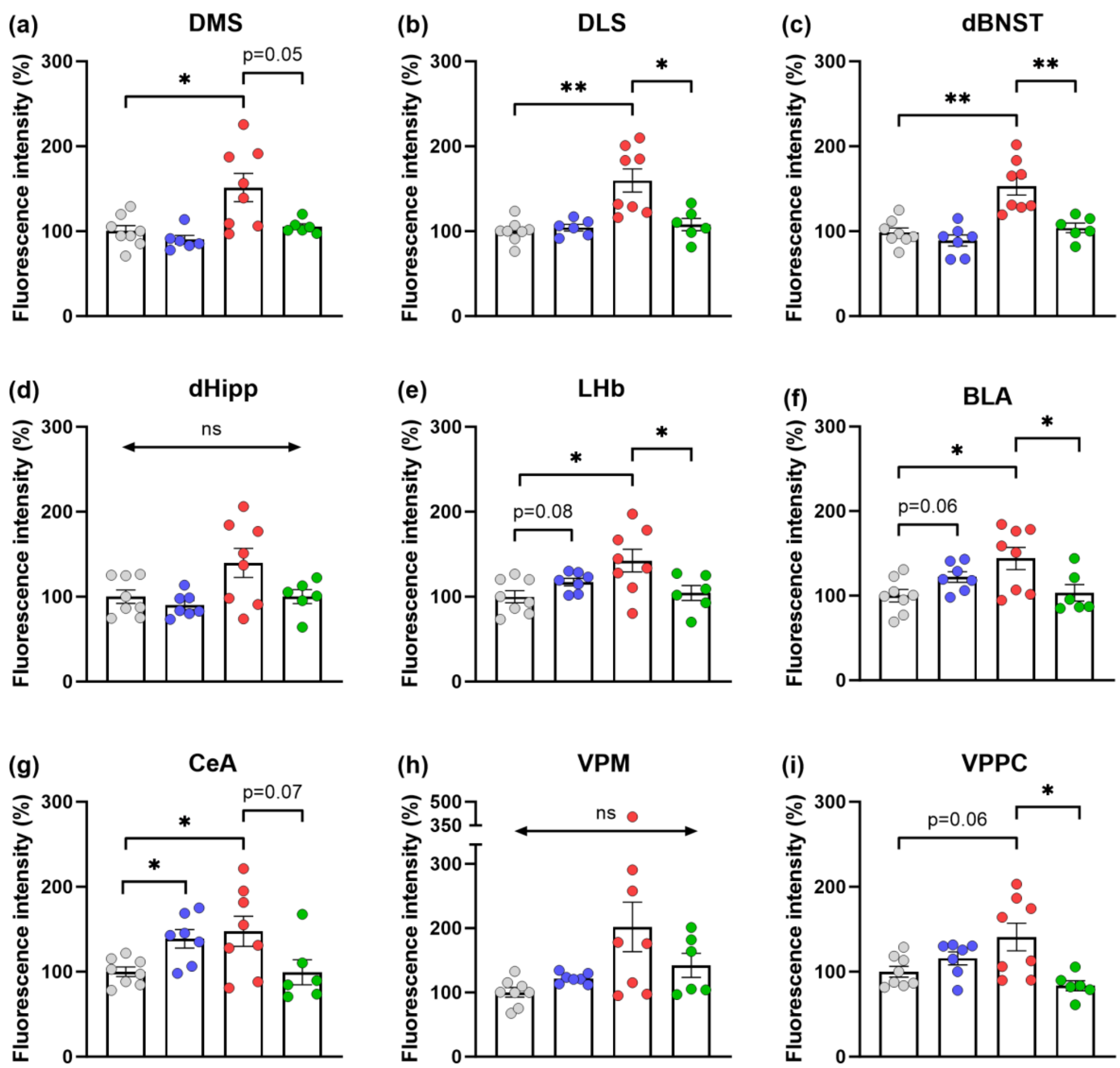

Supplementary data and Figure S4: Relationship between behavioral scores in the light-dark box (LDB) test and IBA-1 relative expression in the studied brain regions.

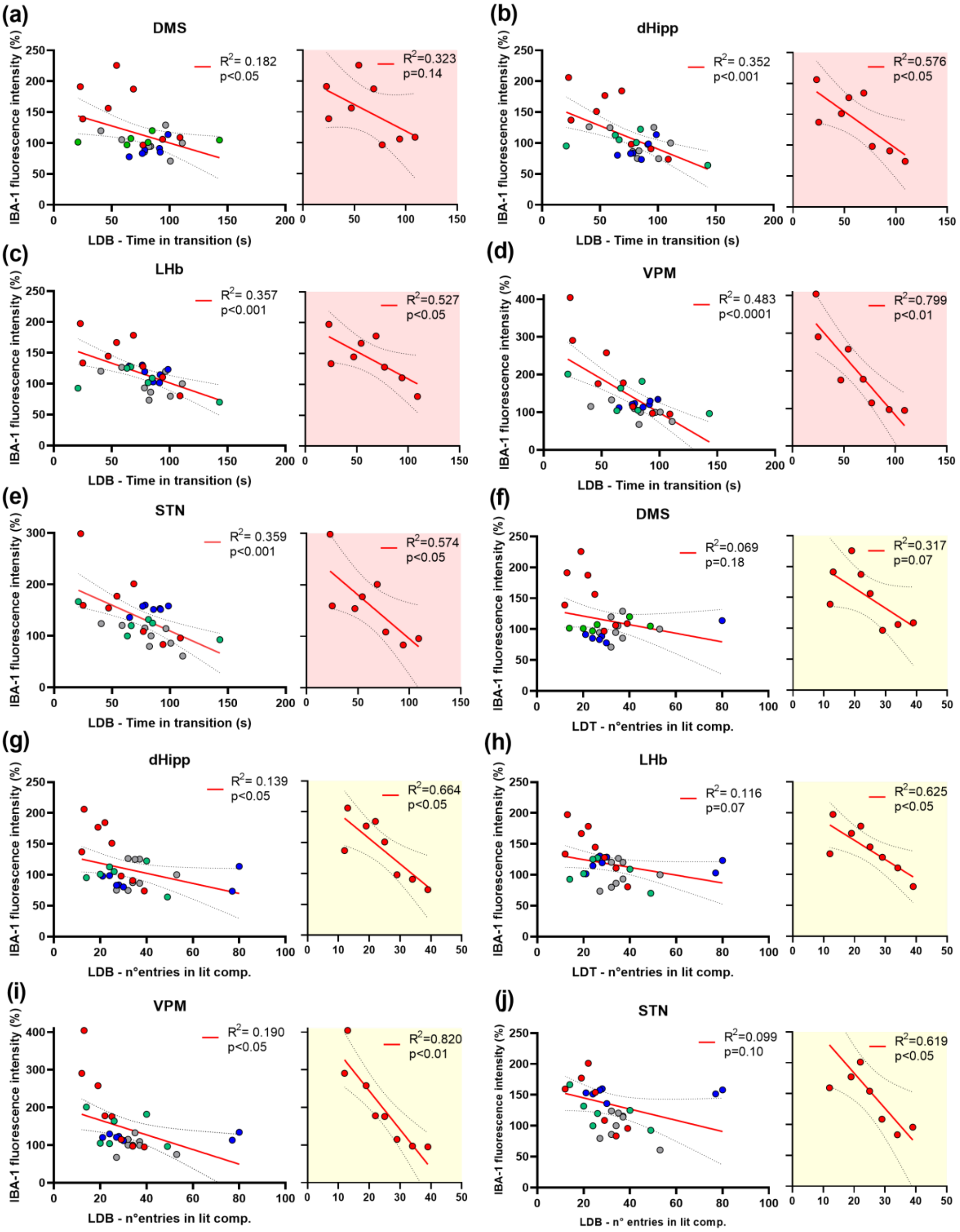

From the overall results, the correlations studies revealed five main anatomical regions (dorsomedial striatum – DMS, dorsal hippocampus – dHipp, lateral habenula – LHb, ventral posteromedial thalamic nucleus – VPM, and subthalamic nucleus of the hypothalamus – STN) that might explain two behavioral scores in the LDB test, i.e., time in the transition zone **(a–e**, white plotting area**)** and number of entries into the lit compartment **(f–j**, white plotting area**)**. However, the variation in the data set

explained by the linear models is globally poor (<50%). Besides, when the untreated pathological condition is isolated (HFD-VEH), the explained variation of the linear models notably increases **(a–e**, time in transition: red plotting area; **f–j**, entries into light: yellow plotting area**)**. The results suggest that, in pathological conditions, IBA-1 expression might account for behavioral variability in the LDB test in the highlighted regions. Color code: grey for STD-VEH, blue for STD-LA, red for HFD-VEH, and green for HFD-LA.

Otherwise, only three other significant correlations were evidenced. For the total time of grooming in the ST, overall scores significantly correlate with IBA-1 expression in the DMS [r=-0.401, p<0.05], but not for HFD-VEH if analyzed separately [r=-0.512, p=0.20]. For the latency of approximation in the NSF task, overall scores significantly correlate with IBA-1 expression in the PSTN [r=0.513, p<0.01], but not for HFD-VEH mice separately [r=0.384, p=0.40]. However, NSF latencies significantly correlate with IBA-1 expression in the PLH of the HFD-VEH experimental group [r=0.716, p<0.05]. No other significant correlations were found [all p-values>0.10].

Supplementary Table T1: Diet composition

Composition of the scheme g/100g	Standard Diet		High Fat Diet (60%)	
	g	*Kcal*	*g*	*Kcal*
Carbohydrate	35.00	140.01	35.43	141.73
Protein	18.50	74.01	14.58	58.33
Lipids	4.50	40.49	35.34	318.02
Fibers	4.50	8.98	2.73	5.45
Minerals Ash	6.30	0	3.68	0
Water	31.20	0	8.24	0
Total	100	263.49	100	523.52
